# Levels and correlates of risk factor control in diabetes mellitus –ELSA-Brasil

**DOI:** 10.1186/s13098-022-00961-3

**Published:** 2023-01-05

**Authors:** Bruna Cristine Chwal, Rodrigo Citton Padilha dos Reis, Maria Inês Schmidt, Bruce B. Duncan, Sandhi Maria Barreto, Rosane Harter Griep

**Affiliations:** 1 Postgraduate Program in Epidemiology, Universidade Federal do Rio Grande do Sul, R. Ramiro Barcelos, 2600/518, Porto Alegre, Rio Grande do Sul 90035-003 Brazil; 2 Departamento de Estatística, Universidade Federal do Rio Grande do Sul, Porto Alegre, Rio Grande do Sul Brazil; 3 Hospital de Clínicas de Porto Alegre, R. Ramiro Barcelos, 2600/518, Porto Alegre, Rio Grande do Sul 90035-003 Brazil; 4 Laboratório de Educação em Ambiente e Saúde, Instituto Oswaldo Cruz, Fundação Oswaldo Cruz, Rio de Janeiro, Rio de Janeiro Brazil; 5 Faculdade de Medicina e Hospital das Clínicas/EBSERH, Universidade Federal de Minas Gerais (UFMG), Belo Horizonte, Minas Gerais Brazil

**Keywords:** Diabetes mellitus, Cardiometabolic risk factors, Glycated hemoglobina A, Hypertension, Hypercholeserolemia, Tobacco smoking

## Abstract

**Background:**

Control of glucose, blood pressure, cholesterol, and smoking improves the prognosis of individuals with diabetes mellitus. Our objective was to assess the level of control of these risk factors in Brazilian adults with known diabetes and evaluate correlates of target achievement.

**Methods:**

Cross-sectional sample of the Brazilian Longitudinal Study of Adult Health, composed of participants reporting a previous diagnosis of diabetes or the use oof antidiabetic medication. We measured glycated hemoglobin (HbA1c) and LDL-cholesterol at a central laboratory and blood pressure following standardized protocols. We defined HbA1c  < 7% as glucose control (target A); blood pressure  < 140/90 mmHg (or  < 130/80 mmHg in high cardiovascular risk) as blood pressure control (target B), and LDL-c  < 100 mg/dl (or  < 70 mg/dl in high risk) as lipid control (target C), according to the 2022 American Diabetes Association guidelines.

**Results:**

Among 2062 individuals with diabetes, 1364 (66.1%) reached target A, 1596 (77.4%) target B, and 1086 (52.7%) target C; only 590 (28.6%) achieved all three targets. When also considering a non-smoking target, those achieving all targets dropped to 555 (26.9%). Women (PR = 1.13; 95%CI 1.07–1.20), those aged  ≥ 74 (PR = 1.20; 95%CI 1.08–1.34), and those with greater per capita income (e.g., greatest income PR = 1.26; 95%CI 1.10–1.45) were more likely to reach glucose control. Those black (PR = 0.91; 95%CI 0.83–1.00) or with a longer duration of diabetes (e.g., ≥ 10 years PR = 0.43; 95%CI 0.39–0.47) were less likely. Women (PR = 1.05; 95%CI 1.00–1.11) and those with private health insurance (PR = 1.15; 95%CI 1.07–1.23) were more likely to achieve two or more ABC targets; and those black (PR = 0.86; 95%CI 0.79–0.94) and with a longer duration of diabetes (e.g., > 10 years since diabetes diagnosis, PR = 0.68; 95%CI 0.63–0.73) less likely.

**Conclusion:**

Control of ABC targets was poor, notably for LDL-c and especially when considering combined control. Indicators of a disadvantaged social situation were associated with less frequent control.

## Introduction

Diabetes mellitus is a chronic and complex disease that requires continuous medical care. In addition to adequate glycemic control, multifactorial risk reduction is indicated [1]. In clinical trial settings, interventions to control hyperglycemia, hypertension, and hypercholesterolemia, as well as to stop smoking, have been shown to produce sustained benefits in vascular complications, with major reductions in cardiovascular outcomes [2, 3]. Additionally, the risk of dying is only 6% greater in those at or below targets than in individuals without diabetes [4].

Recommended therapeutic targets, also called the ABC goals, usually include (A) glycated hemoglobin (HbA1c) less than 7%, (B) a blood pressure < 140/90 mmHg, and (C) LDL-cholesterol (LDL-c) < 100 mg/dl. Tighter control has also been suggested for those with cardiovascular disease or at high risk of developing it [5]. Additionally, non-smoking is an important goal to be achieved.

Studies demonstrating the control of multiple risk factors in diabetes are scarce in low- and middle-income countries. Based on a small subsample of a probabilistic national Brazilian survey, we demonstrated that target achievement is usually poor, except for non-smoking [6]. However, that study lacked statistical power to assess the factors related to ABC control. Therefore, we sought to analyze the Longitudinal Study of Adult Health (ELSA-Brasil) to explore these associations. This large ongoing occupational cohort study enrolled 15,105 adults in 6 capital cities from 2008 to 2010. We aimed to assess the level of control of blood glucose, blood pressure, lipids, and smoking habits of ELSA-Brasil participants with known diabetes mellitus and to evaluate correlates of target achievement.

## Methods

### Study population and ethics

We conducted a cross-sectional study based on the third clinic visit (2017–2019) of ELSA-Brasil. The study was approved by the research ethics committees of participating institutions, and all participants gave their written informed consent. The ELSA-Brasil cohort enrolled 15,105 in-service or retired civil servants aged 35 to 74 at six public institutions of higher education located in capital cities of the states of Bahia, Espírito Santo, Minas Gerais, Rio de Janeiro, São Paulo, and Rio Grande do Sul [7]. We included all participants returning for the 2017–2019 clinic visit with self-report of and/or pharmacologic treatment for diabetes. We excluded those who did not have all measures necessary to assess whether targets were achieved.

### Measurements

Centrally trained and certified teams conducted standardized interviews and clinical assessments and collected samples for biochemical tests [8]. We obtained data on age, sex, self-declared race, history of a medical diagnosis of diabetes, duration of diabetes, and depressive episodes by questionnaire. Physical activity was obtained by leisure-time physical activity and categorized as [1] high  ≥ 1500 MET-minutes/week, [2] moderate (600–1499 MET-minutes/week, and [3] low (< 600 MET-minutes/week).

Medication use was confirmed by packaging or prescriptions brought to the clinic. Blood pressure was measured three times, and systolic and diastolic blood pressure each was ascertained as the mean of the last two measurements. We assessed weight and height using a standardized protocol and calculated body mass index (BMI) as weight/height^2^ (kg/m^2^).

We obtained blood samples after an overnight (> 8 h) fast and froze and shipped them to a central laboratory for determination. Plasma glucose was measured using the hexokinase method (Cobas c501^®^, Roche Diagnostics), HbA1c by high-pressure chromatography (HPLC—Bio-Rad Laboratories, Hercules, CA, USA), total cholesterol by enzymatic colorimetric method, and triglycerides by glycerol-phosphate peroxidase (Cobas c501^®^, Roche Diagnostics). Low-density cholesterol (LDL-c) was estimated by the Friedewald equation when total triglycerides were  < 400 mg/dl and measured directly when they were  ≥ 400 mg/dl.

Ten-year risk of a major cardiovascular event (myocardial infarction, stroke, or cardiovascular death) was estimated based on age, sex, diabetes, smoking, systolic blood pressure, and total cholesterol according to the WHO Risk Chart Working Group chart for the Tropical Latin America region [9]. We categorized this estimated risk as high (≥ 20%) or low.

In line with the 2022 American Diabetes Association (ADA) guidelines for therapeutic targets [10], we considered glucose control to be adequate when HbA1c was less than 7%. For those without high cardiovascular risk, we considered blood pressure  < 140/90 mmHg and LDL-c < 100 mg/dl as meeting targets [5]; and for those with high risk or clinical cardiovascular disease, < 130/80 mmHg and  < 70 mg/dl, respectively [10].

### Statistical analyses

We described categorical variables as frequencies and percentages, and continuous ones as means and standard deviations (SD). We performed unadjusted statistical testing with the chi-square test for categorical and ANOVA for continuous variables. We analyzed the adjusted associations of socio-demographic and clinical factors with the level of control using Poisson regression with robust variance. We undertook all analyses with R software (RStudio, version 1.3. 1056, ^©^2009–2020 RStudio Inc.).

## Results

Of the 15,105 participants at baseline, 550 had died, 123 had moved away, 217 were not localized, 39 were too ill to attend clinic at visit 3 and 1540 did not attend this visit for other reasons. Of the remaining 12,636 (83.7%) participants at visit 3, 2385 (18.9%) had a known diagnosis of diabetes. After excluding 323 participants with missing data on risk factor control or covariates, 2062 remained.

Among these 2062 participants, 1000 (48.5%) were men, 1217 (59.0%) aged 45–64, 961 (46.6%) with self-declared white race/skin color, and 1068 (51.8%) with a complete university education. Additionally, 1380 (66.9%) reported having private health insurance. Mean BMI was 29.6 (4.97) kg/m^2^, and 1812 (87.9%) individuals related use of an antidiabetic drug. (Table 1).

**Table 1:** Sociodemographic and clinical characteristics of individuals with known diabetes according to levels of glycated hemoglobin (HbA1c)—ELSA-Brasil, 2017–2019. N = 2062

	HbA1c					
	Overall	< 7%	7% to < 8%	8% to < 9%	≥ 9%	P*
All	2062 (100)	1364 (66.1)	275 (13.3)	172 (8.34)	251 (12.1)	
Sex						< 0.001
Men	1000 (48.5)	616 (45.2)	157 (57.1)	94 (54.7)	133 (53.0)	
Women	1062 (51.5)	748 (54.8)	118 (42.9)	78 (45.3)	118 (47.0)	
Age (years), M(SD)						0.003
> 44 to ≤ 54	377 (18.3)	256 (18.8)	37 (13.5)	25 (14.5)	59 (23.5)	
> 54 to ≤ 64	840 (40.7)	549 (40.2)	104 (37.8)	77 (44.8)	110 (43.8)	
> 64 to ≤ 74	659 (32.0)	422 (30.9)	108 (39.3)	58 (33.7)	71 (28.3)	
≥ 74	186 (9.02)	137 (10.0)	26 (9.45)	12 (6.98)	11 (4.38)	
Race						< 0.001
Black	423 (20.5)	235 (17.2)	74 (26.9)	33 (19.2)	81 (32.3)	
Pardo	583 (28.3)	378 (27.7)	71 (25.8)	59 (34.3)	75 (29.9)	
White	961 (46.6)	682 (50.0)	117 (42.5)	73 (42.4)	89 (35.5)	
Yellow/Indigenous	95 (4.61)	69 (5.06)	13 (4.73)	7 (4.07)	6 (2.39)	
Education						< 0.001
Less than University	994 (48.2)	562 (41.2)	156 (56.7)	109 (63.4)	167 (66.5)	
University	1068 (51.8)	802 (58.8)	119 (43.3)	63 (36.6)	84 (33.5)	
Private health insurance						< 0.001
Yes	1380 (66.9)	972 (71.3)	172 (62.5)	112 (65.1)	124 (49.4)	
Per capita income (minimum wages/month)^1^						< 0.001
Less than 4	356 (17.3)	174 (12.8)	71 (25.8)	36 (20.9)	75 (29.9)	
From 4 to less than 8	760 (36.9)	489 (35.9)	89 (32.4)	72 (41.9)	110 (43.8)	
From 8 to less than 12	375 (18.2)	250 (18.3)	55 (20.0)	33 (19.2)	37 (14.7)	
From 12 to less than 16	214 (10.4)	171 (12.5)	21 (7.64)	11 (6.40)	11 (4.38)	
16 or more	357 (17.3)	280 (20.5)	39 (14.2)	20 (11.6)	18 (7.17)	
BMI (kg/m^2^), M (SD)	29.6 (4.97)	29.5 (4.97)	29.8 (5.23)	29.5 (4.37)	29.8 (5.09)	0.671
Diabetes medication						0.521
Yes	1812 (87.9)	1201 (88.0)	238 (86.5)	156 (90.7)	217 (86.5)	
WHO CVD risk						
High CVD risk	53 (2.57)	28 (2.05)	7 (2.55)	8 (4.65)	10 (3.98)	0.084
Years since diabetes diagnosis						< 0.001
0–1	352 (17.1)	346 (25.4)	1 (0.36)	2 (1.16)	3 (1.20)	
1–10	1062 (51.5)	755 (55.4)	133 (48.4)	64 (37.2)	110 (43.8)	
10+	648 (31.4)	263 (19.3)	141 (51.3)	106 (61.6)	138 (55.0)	

n (%) unless otherwise indicated. Except for the row “All”, percentages are for column totals. Yellow skin color refers to Asian ancestry

*M (SD)* mean (standard deviation)

^*^Chi-square test for categorical variables and ANOVA for continuous ones of statistical significance of the difference in variable level or frequency across categories of HbA1c. ^1^SM = The monthly minimum wage was BRL 986.00 at the time of the study

**Fig. 1:**
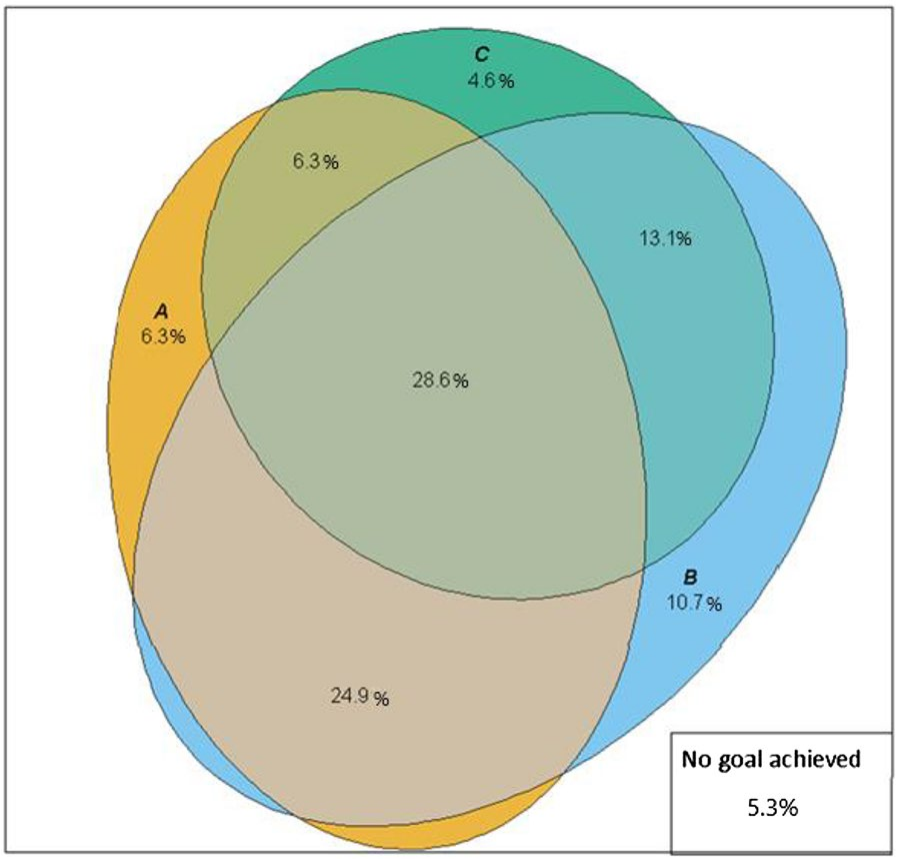
Venn diagram showing the overlap in the attainment of treatment goals in individuals with self-reported diabetes: HbA1c (**A**), blood pressure (**B**), and LDL-c (**C**). ELSA-Brasil, 2017–2019. N = 2062

**Fig. 2:**
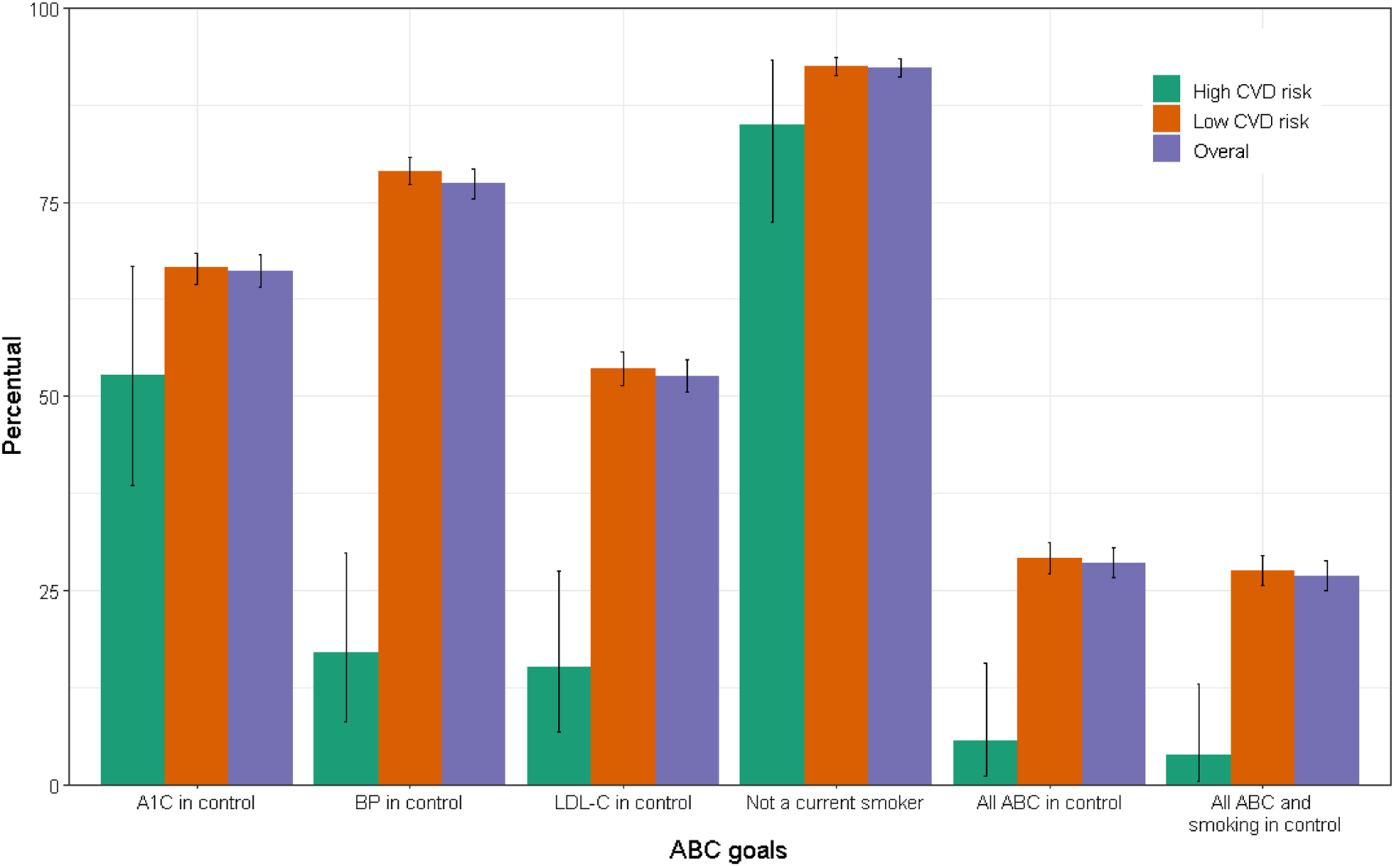
Percentual of attainment of each of the three treatment goals, with 95% confidence interval, of non-smoking and of combinations of the goals among individuals with self-reported diabetes in Elsa-Brasil, 2017–2019. N = 2062

Figure 1, which presents the overlap in achieving goals, shows greater success in achieving that of blood pressure and lesser success in achieving that of LDL-c, as well as no particular pattern of clustering in the success of achieving more than one goal. Only 589 (28.6%) achieved all three goals (center of the figure). As shown in Figs. 1, 2, HbA1c was at or below target in 1364 (66.1%), blood pressure in 1596 (77.4%), and LDL-c in 1086 (52.7%). Control was more frequent in those without high risk for CVD, reflecting the more rigorous targets for those at high risk. Non-smokers comprised 1904 (92.3%) of the sample. However, all ABCs were at or below target in only 590 (28.6%) participants and, when also considering non-smoking as a target, in 555 (26.9%).

As further seen in Table 1, in crude comparisons, being a woman, older, white, and having greater educational attainment, greater income, and private health insurance, as well as having a shorter duration of diabetes were all associated with better glucose control.

As seen in crude comparisons in Table 2, several sociodemographic and clinical characteristics, mostly the same as those seen in Table 1, were associated with a greater number of ABC goals being reached—being white, having greater educational attainment, greater income, and private health insurance, pursuing greater physical activity, having a lower BMI and using antidiabetic medications.

**Table 2:** Socio-demographics and clinical characteristics of individuals with type 2 diabetes according to the number of ABC goals reached. ELSA-Brasil, 2017–2019. N = 2062

		Goals reached			
	Overall	0	1 or 2	3	p*
All	2062(100)	111 (5.4)	1360 (66.0)	589(28.6)	
Sex					
Men	1000 (48.5)	60 (54.1)	655 (48.1)	285 (48.3)	0.483
Women	1062 (51.5)	51 (45.9)	706 (51.9)	305 (51.7)	
Age (years), M (SD)					0.002
> 44 to ≤ 54	377 (18.3)	13 (11.7)	273 (20.1)	91 (15.4)	
> 54 to ≤ 64	840 (40.7)	44 (39.6)	568 (41.7)	228 (38.6)	
> 64 to ≤ 74	659 (32.0)	47 (42.3)	407 (29.9)	205 (34.7)	
≥ 74	186 (9.02)	7 (6.31)	113 (8.30)	66 (11.2)	
Race					< 0.001
Black	423 (20.5)	38 (34.2)	303 (22.3)	82 (13.9)	
* Pardo* (mixed)	583 (28.3)	36 (32.4)	389 (28.6)	158 (26.8)	
White	961 (46.6)	33 (29.7)	604 (44.4)	324 (54.9)	
Yellow/Indigenous	95 (4.61)	4 (3.60)	65 (4.78)	26 (4.41)	
Education					< 0.001
Less than University	994 (48.2)	81 (73.0)	701 (51.5)	212 (35.9)	
University	1068 (51.8)	30 (27.0)	660 (48.5)	378 (64.1)	
Private health insurance Yes	1380 (66.9)	48 (43.2)	876 (64.4)	456 (77.3)	< 0.001
Per capita income (minimum wages/month)^1^					< 0.001
Less than 4	356 (17.3)	37 (33.3)	260 (19.1)	59 (10.0)	
From 4 to less than 8	760 (36.9)	43 (38.7)	518 (38.1)	199 (33.7)	
From 8 to less than 12	375 (18.2)	17 (15.3)	247 (18.1)	111 (18.8)	
From 12 to less than 16	214 (10.4)	9 (8.11)	127 (9.33)	78 (13.2)	
16 or more	357 (17.3)	5 (4.50)	209 (15.4)	143 (24.2)	
BMI (kg/m^2^), M (SD)	29.6 (4.97)	30.3 (5.48)	29.5 (4.89)	29.6 (5.05)	0.245
Physical activity (MET-minutes/week)^2^					0.046
Low	1490 (72.3)	89 (80.2)	995 (73.1)	406 (68.8)	
Moderate	453 (22.0)	20 (18.0)	292 (21.5)	141 (23.9)	
High	119 (5.77)	2 (1.80)	74 (5.44)	43 (7.29)	
Depressive episodes					
Yes	107 (5.19)	6 (5.41)	74 (5.44)	27 (4.58)	0.729
Diabetes medication					< 0.001
Yes	1805 (87.5)	91 (82.0)	1172 (86.1)	541 (91.7)	
Years since diabetes diagnosis					< 0.001
0–1	352 (17.1)	2 (1.80)	199 (14.6)	151 (25.6)	
1–10	1062 (51.5)	54 (48.6)	699 (51.4)	309 (52.4)	
10 +	648 (31.4)	55 (49.5)	463 (34.0)	130 (22.0)	

n (%) unless otherwise indicated. Except for the row “All”, percentages are for column totals. Yellow skin color refers to Asian ancestry, physical activity refers to physical activity during leisure time.

*M (SD)* mean (standard deviation)

^*^Chi-square test for categorical variables and ANOVA for continuous ones of statistical significance of the difference in variable level or frequency across categories of HbA1c

^*1*^SM = *The monthly minimum wage was BRL 986.00 at the time of the study*

^*2*^*High 1500 MET-minutes*/week*; moderate 600 MET-minutes*/week; low less than 600 MET minutes/week

As seen in Table 3, after adjustment for multiple potential confounders, women (PR = 1.13; 95%CI 1.07–1.20), those  ≥ 74 years old (RR = 1.20; 95%CI 1.08–1.34), and those with greater income (e.g. for those with the highest income (PR 1.26. 95%CI 1.10–1.45) were more likely to achieve the HbA1c goal, while those black (PR = 0.91; 95%CI 0.83–1.00) and with diabetes of longer duration (e.g. ≥ 10 years PR = 0.43; 95%CI 0.39–0.47) less likely. In terms of ABC goals, women (PR = 1.05; 95%CI 1.00–1.11) and those with private health insurance (PR = 1.15; 95%CI 1.07–1.23) and with higher per capita income (e.g. for those with highest income PR = 1.19; 95%CI1.06–1.34) had a greater probability of meeting two or more ABC goals. On the other hand, those black (PR = 0.86; 95%CI 0.79–0.94), with greater body mass index (PR = 0.96; 95%CI 0.94–0.99), and with a longer duration of diabetes (e.g. ≥ 10 years PR = 0.68; 95%CI 0.63–0.73) were less likely to achieve two or more of the goals.

**Table 3:** Adjusted* associations of selected population and clinical characteristics among individuals with known type 2 diabetes. ELSA-Brasil, 2017–2019. N = 2062

Characteristic	HbA1C < 7%		Achieving ≥ 2 treatment goals**	
	PR (95% CI)	P	PR (95% CI)	P
Sex (reference: men)				
Women	1.13 (1.07–1.20)	< 0.001	1.05 (1.00–1.11)	0.050
Age (years, reference: ≤ 54)				
> 54 to ≤ 64	1.03 (0.95–1.11)	0.453	1.01 (0.94–1.08)	0.836
> 64 to ≤ 74	1.08 (0.99–1.17)	0.080	1.03 (0.96–1.11)	0.415
≥ 74	1.20 (1.08–1.34)	< 0.001	1.02 (0.91–1.04)	0.646
Race (reference: white)				
Black	0.91 (0.83–1.00)	0.041	0.86 (0.79–0.94)	< 0.001
*Pardo (mixed)*	1.01 (0.95–1.08)	0.701	0.95 (0.90–1.02)	0.139
Yellow/indigenous	1.05 (0.93–1.19)	0.433	0.94 (0.83–1.06)	0.307
Education (reference: university)				
Less than university	0.94 (0.86–1.01)	0.101	0.97 (0.91–1.04)	0.419
Private health insurance (reference: no)				
Yes	1.07 (0.99–1.15)	0.083	1.15 (1.07–1.23)	< 0.001
Per capita income (minimum wages/mo. Reference: less than 4)^1^				
4 to < 8	1.20 (1.07–1.34)	0.002	1.12 (1.01–1.23)	0.031
8 to < 12	1.16 (1.02–1.32)	0.023	1.11 (1.00–1.25)	0.061
12 to < 16	1.27 (1.11–1.45)	0.001	1.18 (1.05–1.33)	0.007
≥ 16	1.26 (1.10–1.45)	0.001	1.19 (1.06–1.34)	0.004
Body mass index (increase of 5 kg/m^2^)	0.97 (0.95–1.00)	0.087	0.96 (0.94–0.99)	0.012
Diabetes medication				
Yes	1.03 (0.94–1.12)	0.535	1.03 (0.95–1.12)	0.507
Years since diabetes diagnosis (reference: 0–1)				
1–10	0.74 (0.71–0.77)	< 0.001	0.87 (0.82–0.91)	0.001
10 +	0.43 (0.39–0.47)	< 0.001	0.68 (0.63–0.73)	< 0.001

^*^through Poisson regression with robust variance for age, sex, educational achievement, race/skin color, private health insurance, per capita income, diabetes medication, body mass index, and years since diagnosis

^**^Treatment goals: glucose, blood pressure and LDL-c

^1^Minimum wage: the monthly minimum wage was BRL 986.00 at the time of the study

## Discussion

In this free-living sample of 2063 Brazilian adults with known diabetes, HbA1c was controlled in more than half of the sample (66.1%), as were blood pressure (77.4%) and LDL-c (52.6%). However, only 28.6% of participants had all three factors controlled. Some indicators of greater social privilege (white ethnicity, higher income, and access to private health insurance) are associated with meeting targets.

The fraction of individuals reaching glycemic, blood pressure, and LDL-c goals in the ELSA-Brasil cohort was greater than that seen in the 2013 Brazilian National Health Survey [6]: 66.1% in ELSA-Brasil vs 46% in the national survey when using identical control cutoffs for targets. Attainment of all three ABC goals in ELSA-Brasil participants was also greater than in this Brazilian National Health Survey (28.6% vs 12.5%). Consonant with the high estimates of non-smoking in Brazilian adults in general, achievement of the non-smoking target was similar in both studies (92.3% vs 90.3%) and higher than those found in other surveys [11, 12]. This achievement results from the long-term implementation of multiple, strong public policies against tobacco [13] in Brazil.

In studies in diverse countries, attainment of all ABC goals was always low. In the US NHANES, 22.2% of individuals simultaneously achieved all three targets (HbA1c  < 7%, blood pressure  < 140/90 mmHg, and non–high-density lipoprotein cholesterol  < 130 mg/dl) [14]. In the Korean NHANES, with a more stringent target for glycemic control (HbA1c < 6.5%), and with targets of blood pressure  < 140/85 mmHg, and LDL-C below 100 mg/dl, only 8.4% of subjects reached all three targets [15]. In a study in nine Latin American countries, glycemic control was also lower (43.5%) than that described here [16]. Our findings thus complement those already present in the literature, showing the current difficulty faced by diabetic patients in achieving desired levels of the principal factors affecting their prognosis which are modifiable at the individual level.

We found several characteristics that identified those not reaching targets for hyperglycemia and the ABC goals. As expected, a greater duration of diabetes was one. A higher BMI was marginally associated with a lesser frequency of control. Women were more frequently in glycemic control and achieved greater ABC control. The additional factors associated with worse control—being non-white, with lower income, and not having private health insurance, all point to better control being in part the result of social privilege.

Similarly, a representative survey of adults with diabetes showed that private health insurance led to their receiving better quality primary care, as measured by the cardinal attributes of quality primary care, especially access. Greater access to care provides a logical pathway linking this insurance to better control [17]. A multicenter Brazilian study of hospital outpatients showed that multi-professional care and having had diabetes education as well as disease of lesser duration significantly associated with improved glycemic control [18]. Morães et al., evaluating only glycemic control at the baseline ELSA-Brasil visit, demonstrated similar associations with socioeconomic factors [19] as those we found here for overall ABC target achievement.

Our study has limitations, principally that our sample is composed of active or retired civil servants, a socially privileged sample when compared to the general Brazilian population in terms of educational achievement, income, and job stability among other factors. That we found major socioeconomic determinants of control in this more privileged population only emphasizes the likelihood of greater health disparities in achieving ABC targets in the general diabetic population.

Strengths of our study include its free-living sample of participants obtained in multiple cities across Brazil, different from many other studies which investigated less representative inpatient or outpatient samples which will have both more comorbidities and, by their entry criteria, better access to care. Additional strengths include ELSA´s careful and extensive collection of factors examined, its standardized and centralized laboratory measurements, and its sample size permitting adequate investigation of epidemiologically relevant associations.

As has been shown for health outcomes in general [20], the correlates of control we found demonstrate the major role of social determinants of health in the ABCs of diabetes control. As put forth by the American Heart Association, clinical care and treatment account for 10% to 20% of the modifiable contributors to health outcomes. The other 80% to 90% are the social determinants of health, which include health-related behaviors, socioeconomic factors, environmental factors, and racism, all recognized to have a profound impact on cardiovascular disease and diabetes and their outcomes [21]. The ADA also recently summarized what is known about the importance of social determinants [22]. One implication from these findings is clear: though better control across the board is necessary, improvement and greater resources for the care for people with diabetes in the SUS, Brazil´s national health system should be a major goal if the aim is to improve control in the overall population of those with diabetes in Brazil. The SUS covers the bulk of the population and the majority of its underprivileged citizens. It also presents the advantage of providing cost-effective, evidence-based protocols to achieve treatment goals.

These findings are particularly relevant now, as actions aimed to achieve greater control at the health system level, supported by greater tracking and feedback of care, are now feasible given advances in information technology. The implementation and expansion of a diabetes registry orienting patient care in several Asian countries produced improvement in control of all the ABCs. In Hong Kong, a setting for which longer follow-up is available, the implementation of the registry was accompanied by a 40% decrease in CVD or microvascular complications and a 66% decrease in all-cause mortality, [23] and was additionally estimated to be cost-saving. [24] In Brazil, advances in the integration of databases within the national health system, which favors primary care and focuses resources on underprivileged communities, offer great hope in this regard. In this scenario, our study, by expanding knowledge of control of diabetes in Brazil and demonstrating the major role of socioeconomic factors, contributes to future strategies for better control and health promotion of Brazilians with diabetes. Future research can refine questions related to the relative benefit of greater control across the board as opposed to focus on better control among those with worst baseline levels.

Additionally, issues of relaxed control, especially of HbA1c, in older patients and those with greater morbidity and thus greater difficulty in managing multiple medications, are also important [5]. The American Heart Association currently emphasizes a comprehensive approach to the management of all cardiovascular risk factors in patients with diabetes, including glycemic, blood pressure, lipid abnormalities, thrombotic risk, obesity, and smoking through applying lifestyle and pharmacological approaches with proven benefit using a patient-centered approach. This latter implies reframing clinical encounters to approach patients as people who live in families, communities, and societies that must be considered in their cardiovascular risk management. [5] While the ideal fraction of the diabetes population in control of all the ABCs, given these issues, is a question that remains open for debate, certainly it is much greater than the current fraction.

In conclusion, control of ABC targets was poor, notably for LDL-c and especially when considering combined control. Our findings reinforce that much room exists for improvement in controlling these modifiable prognostic factors, notably LDL-c and especially when considering combined control. Less frequent control among those black, with lower income, and without health insurance reinforces the role of social factors in the multicausal context of control of risk factors for complications in diabetes. With due attention to social determinants and focusing on better integration of health system data to evaluate and orient patient care, health systems and clinicians can and should strive to implement better care for people with diabetes.

## Data Availability

Due to ethical restrictions approved by the ethics committee of each institution (Universidade Federal da Bahia, Universidade Federal de Minas Gerais, Universidade Federal do Espírito Santo, Fundação Oswaldo Cruz, Universidade de São Paulo, Universidade Federal do Rio Grande do Sul) and by the Publications Committee of ELSA-Brasil (publiELSA), the data used in this study can be made available for research proposals by a request to ELSA’s Datacenter (estatisticaelsa@gmail.com) and to the ELSA’s Publications Committee.
